# A Novel Platinum Resistance-Related Immune Gene Signature for Overall Survival Prediction in Patients with Ovarian Cancer

**DOI:** 10.1007/s10528-023-10379-9

**Published:** 2023-06-04

**Authors:** Chenfei Zhou, Junnan Ma, Wanjun Luo, Jiemei Hu, Jing Chen, Suiying Liang, Shanyang He

**Affiliations:** 1 Department of Gynecology, Guangdong Provincial People’s Hospital (Guangdong Academy of Medical Sciences), Southern Medical University, Guangzhou, 510080 Guangdong People’s Republic of China; 2 Guangdong Provincial People’s Hospital, School of Medical, South China University of Technology, Guangzhou, 510080 Guangdong People’s Republic of China; 3 Guangdong Cardiovascular Institute, Guangzhou, 510080 Guangdong People’s Republic of China; 4 Department of Obstetrics and Gynecology, The First Affiliated Hospital, Sun Yat-Sen University, 510080 Guangzhou, Guangdong People’s Republic of China

## Abstract

Ovarian cancer (OV) is a highly heterogeneous gynecological tumor that makes the prognostic prediction challenging. Resistance to platinum-based chemotherapy is associated with a poor prognosis in OV. There seems to be an overlap between molecular mechanisms responsible for platinum resistance and immunogenicity in OV. However, the predictive role of platinum resistance-related immune genes for OV prognosis needs to be further explored. In our study, the mRNA expression data of OV patients with corresponding clinical information were collected from The Cancer Genome Atlas (TCGA) cohort and International Cancer Genome Consortium (ICGC) cohort. A multigene signature was constructed for OV patients in the TCGA cohort using the least absolute shrinkage and selection operator (LASSO) Cox regression model according to the optimal value of λ and was validated in the ICGC cohort. Furthermore, we performed functional analysis to explore the immune status between low- and high-risk groups based on the median value of the risk score for the multigene signature. Our data showed that there were 41.1% of the platinum resistance-related genes which differentially expressed between immune score low- and high-OV patients in the TCGA cohort. Univariate Cox regression analysis identified 30 differentially expressed genes (DEGs) associated with overall survival (OS) (*P* < 0.05). 14 genes were identified to construct a novel platinum resistance-related immune model for classifying OV patients into the low- and high- risk groups. Patients in the low-risk group showed significantly higher OS than those in the high-risk group (*P* < 0.0001 in the both TCGA and ICGC cohort), which was associated with different immune status for the two risk groups. A novel platinum resistance-related immune model can be used for prognostic prediction in OV. Targeting tumor immunity may be a therapeutic alternative for OV with platinum resistance.

**Supplementary Information:**

The online version contains supplementary material available at 10.1007/s10528-023-10379-9.

## Introduction

Ovarian cancer (OV) is a highly malignant gynecologic tumor in the world. It accounts for 2.5% of female malignant tumors, but 5% of cancer deaths due to low survival rates (Siegel et al. 2021). The standard treatment for OV usually involves a combination of surgery and platinum-based chemotherapy (Torre et al. 2018). Despite treatment availability, the recurrence rate of OV is very high, and for approximately 75% of patients with the disease at advanced stages, recurrence is incurable (Lheureux et al. 2019). The recurrence of OV is closely related to reduced sensitivity to platinum-based antineoplastic drugs. Therefore, it is necessary to further explore platinum resistance related to survival time, which may contribute to the development of more effective treatment methods for OV.

Platinum is demonstrated to induce extensive DNA damage by inducing DNA cross-links, thereby suppressing tumor cell proliferation and enhancing the apoptosis of proliferating cells, which leads to tumor eradication. Primary or acquired resistance to platinum is associated with diverse biological processes, including genetic mutation, alterations of anti-apoptosis signaling pathways, expression of neoplastic antigens, and abnormal DNA damage repair (Li et al. 2020). Moreover, the characteristics of the immunosuppressive tumor microenvironment may also result in platinum resistance in OV (Tian et al. 2022). For example, M2-polarized tumor-associated macrophages (TAMs) suppress the infiltration of lymphocytes into the tumor epithelium, promoting tumors from an “inflamed” phenotype to an “immune-excluded” phenotype (Cummings et al. 2021), which are associated with platinum resistance. Nevertheless, many other aspects of platinum resistance, such as representative prognostic genes in platinum-resistance OV, remain unclear.

Unsurprisingly, there seems to be an inextricable link between platinum resistance and tumor immunity. In OV patients with platinum-resistance disease, markers of an antitumor immune response can be detected in tumor tissue, ascites fluid, and peripheral blood (Santoiemma et al. 2016). A meta-analysis of ten studies with 1815 OV patients demonstated the prognostic value of intraepithelial CD8^+^ tumor-infiltrating lymphocyte (TIL), regardless of tumor grade, stage, or histological subtype although it should be noted that heterogeneity between studies was significant (Hwang et al. 2012). In addition, the tumor-infiltrating B lymphocytes are considered to be associated with improved prognosis in a report by Nielsen et al. (Nielsen et al. 2012). Therefore, platinum-resistance OV patients may present certain characteristics relevant to better benefits from immunotherapy agents, and it is necessary to do more research to understand their relationship.

In this study, mRNA expression profiles and corresponding clinical data were firstly collected from The Cancer Genome Atlas (TCGA) cohort and International Cancer Genome Consortium (ICGC) cohort. Then, a prognostic multigene signature with platinum resistance-related differentially expressed genes (DEGs) was built between the high and low immune score groups in the TCGA cohort and validated it in the ICGC cohort. Finally, the immune functional enrichment analysis was performed to explore the underlying mechanisms.

## Materials and Methods

### Data Collection

The RNA sequencing (RNA-seq) data and corresponding clinical information of 228 OV patients were downloaded from the TCGA website (http://portal.gdc.cancer.gov/). The scale method provided in the “limma” R package was used to normalize the gene expression profiles. RNA-seq data and clinical information of another 82 OV samples were collected from the ICGC cohort (https://dcc.icgc.org/projects/OV-AU). Normalized read count values were used. The data from TCGA and ICGC cohort are both publicly available. The current research follows the TCGA and ICGC data access policies and publication guidelines.

Then, 912 platinum resistance-related genes were downloaded from the database of genes associated with platinum resistance in cancer (http://ptrc-ddr.cptac-data-view.org.) and are provided in Supplementary Tables S1. The immune score of ovarian cancer was retrieved from ESTIMATE (https://bioinformatics.mdanderson.org/estimate/disease.html).

### Construction and Validation of a Prognostic Platinum Resistance-Related Gene Signature

The DEGs between immune score low- and high-OV patients were identified using the “limma” R package with a false discovery rate (FDR) < 0.05 in the TCGA cohort. The platinum resistance-related genes with prognostic values were screened via Univariate Cox analysis of overall survival (OS). *P* values were adjusted by Benjamini & Hochberg (BH) correction. To minimize the risk of overfitting, LASSO-penalized Cox regression analysis was applied to build a prognostic model. The variable selection and shrinkage using the LASSO algorithm in the “glmnet” R package. The independent variable in the regression was the normalized expression matrix of candidate prognostic DEGs, and the response variables were the OS and status of patients in the TCGA cohort. Tenfold cross-validation was used to determine the penalty parameter (*λ*) for the model following the minimum criteria (i.e., the value of λ corresponding to the lowest partial likelihood deviance). The risk scores of the patients were calculated based on the normalized expression level of each gene and its corresponding regression coefficients. The formula was established as follows: score = e ^sum (each gene’s expression * corresponding coefficient)^. The OV patients were stratified into high- and low-risk groups according to the median risk score. The optimal cut-off expression value was calculated via the “surv_cutpoint” function of the “survminer” R package for the survival analysis of every selected gene. The predictive power of the gene signature was evaluated using the time-dependent ROC curve analysis in the “survival ROC” R package.

### Immune Status Analysis

The single-sample gene set enrichment analysis (ssGSEA) was used to calculate the infiltrating score of 16 immune cells and the activity of 13 immune-related pathways by the “gsva” R package. The annotated gene set file is provided in Supplementary Table S2.

### Statistical Analysis

Gene expression between immune score low- and high-OV patients was compared by Student’s t test. Chi-squared test was used to compare the differences in proportions. ssGSEA scores of immune cells or pathways between the high- and low-risk groups were compared using the Mann–Whitney test with *P* values adjusted by the BH method. The OS between different groups was compared by Kaplan–Meier analyses with the log-rank test. The independent predictors of OS was identified by the univariate and multivariate Cox regression analysis. R software (Version 3.5.3) or SPSS (Version 23.0) was used to perform all statistical analyses. *P* < 0.05 was considered statistically significant.

## Results

**Fig. 1:**
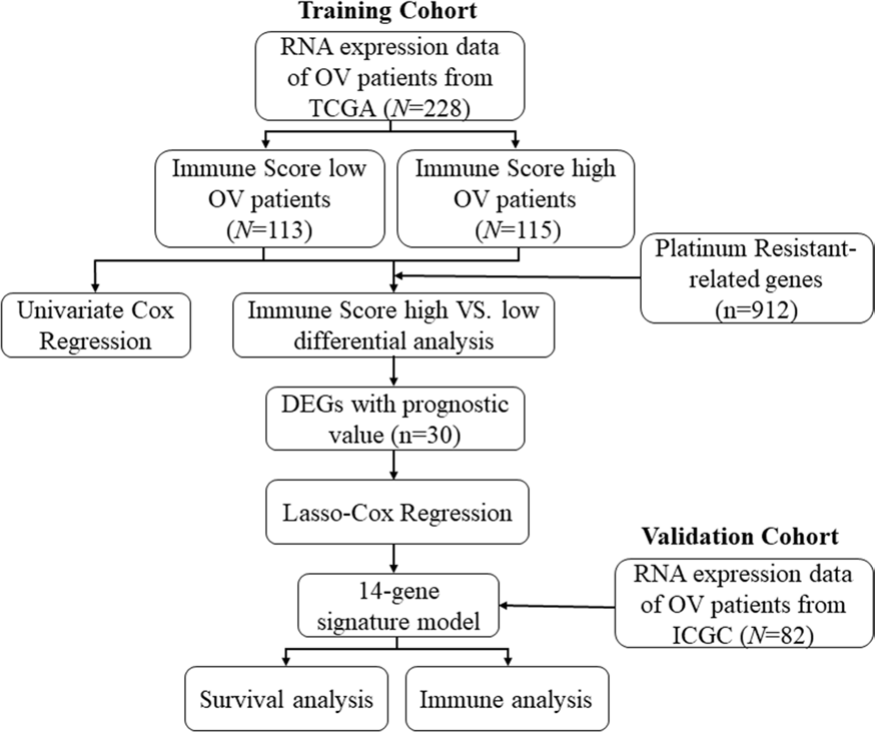
Flow chart of data collection and analysis

The detailed workflow of this study is shown in Fig. 1. A total of 228 OV patients from the TCGA-OV cohort and 82 OV patients from the ICGC (OV-AU) cohort were finally collected. The detailed clinical characteristics of these patients are summarized in Table 1.

**Table 1:** Clinical characteristics of the OV patients used in this study

	TCGA Cohort	ICGC Cohort
No. of patients	228	82
Age (median, range)	58 (34–87)	59 (39–78)
Grade (%)		
Grade 1	1 (0.4%)	NA
Grade 2	26 (11.4%)	NA
Grade 3	193 (84.6%)	NA
Grade 4	1 (0.4%)	NA
unknow	7 (3.1%)	NA
Stage (%)		
I	0 (0.0%)	0 (0.0%)
II	16 (7.0%)	0 (0.0%)
III	181 (79.4%)	69 (84.1%)
IV	29 (12.7%)	13 (15.9%)
unknow	2 (0.8%)	0 (0.0%)
Survival status		
OS days (median)	887 (9–5481)	804 (133–6139)
Censored (%)	37 (16.2%)	11 (13.4%)

### Identification of Prognostic Platinum Resistance-Related Immune DEGs in the TCGA Cohort

**Fig. 2:**
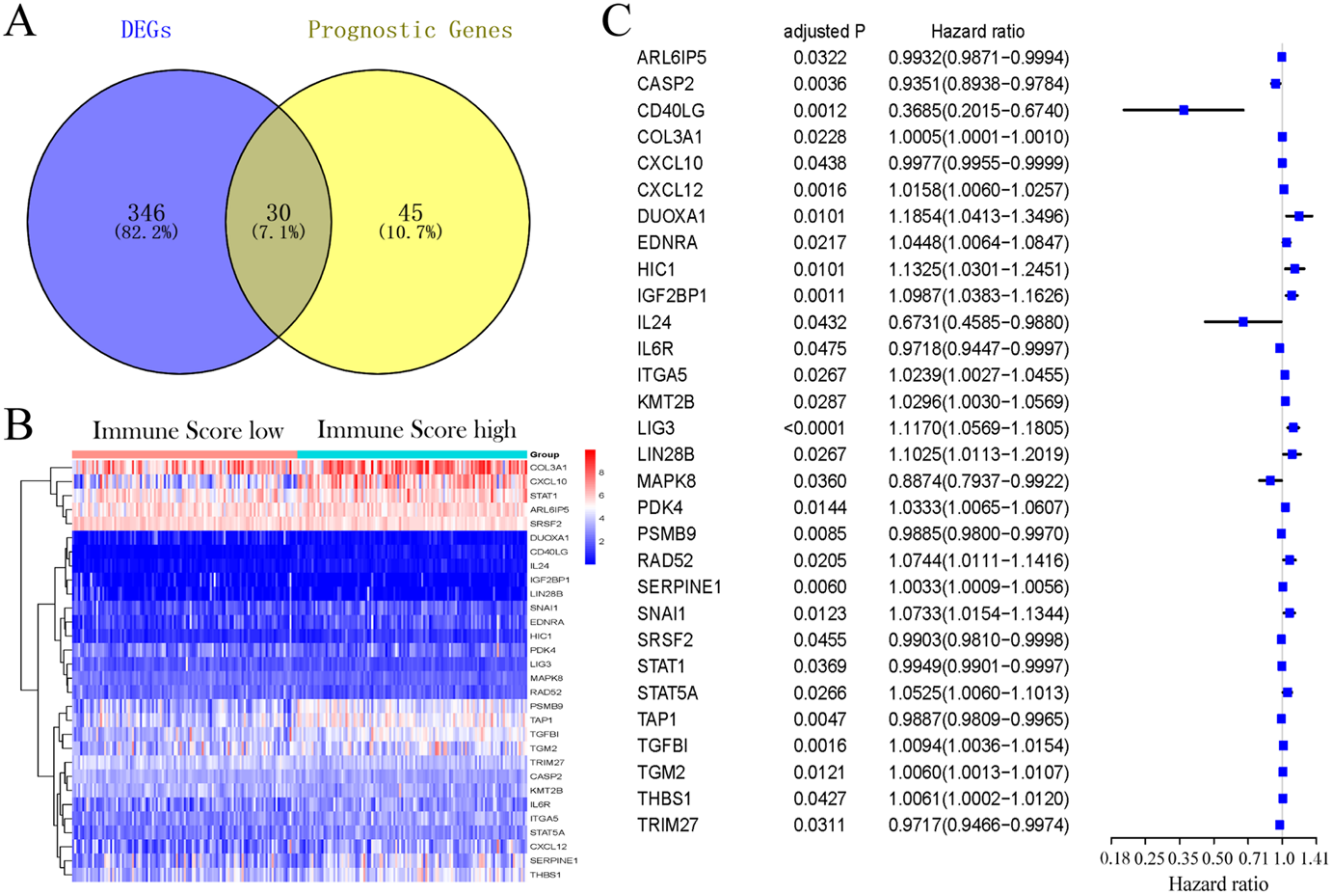
Identification of the prognostic platinum resistance-related immune genes in the TCGA cohort. **A** Venn diagram to identify differentially expressed genes with immune score that were correlated with OS. **B** The 30 overlapping genes were all upregulated in tumor tissue. **C** Forest plots showing the results of the univariate Cox regression analysis between gene expression and OS

Some of the platinum resistance-related genes (376/912, 41.1%) were differentially expressed according to the immune score, and 30 of them were correlated with OS in the univariate Cox regression analysis (Fig. 2A). The heatmap showed that samples between the high and low immune score groups could be distinguished by the 30 prognostic platinum resistance-related DEGs (Fig. 2B). The univariate Cox regression analysis between the expression of these genes (*P* < 0.05) and OS is shown in forest plots (Fig. 2C).

### Construction of a 14-Gene Prognostic Model in the TCGA Cohort and Validation of the Model in the ICGC Cohort

**Fig. 3:**
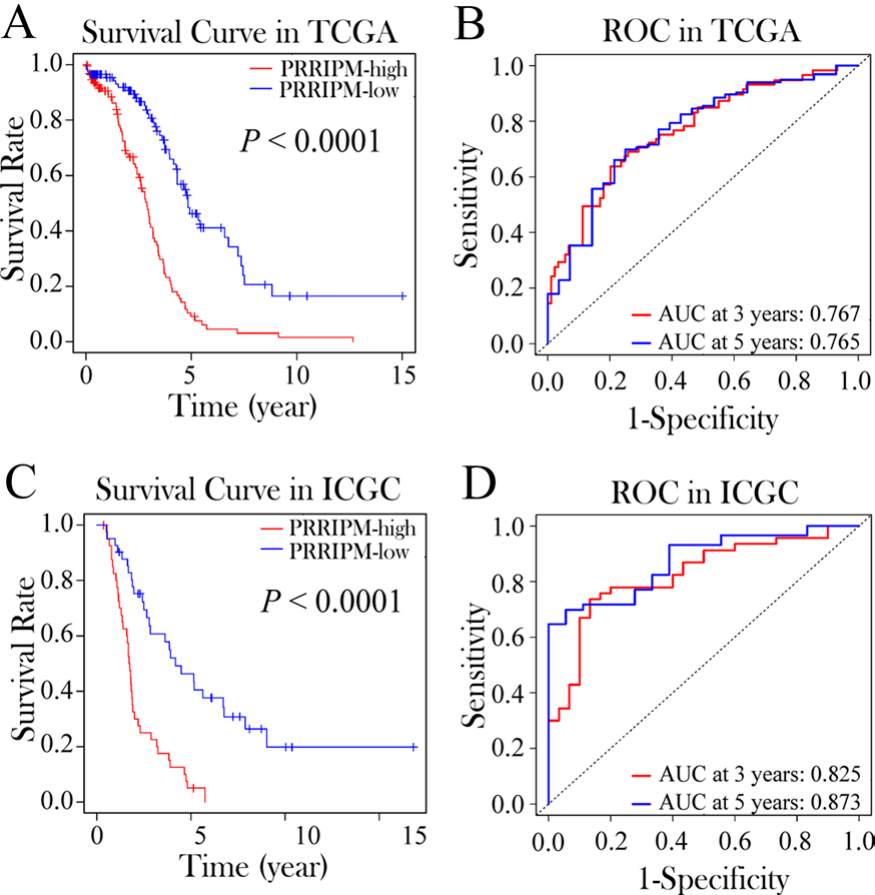
Prognostic analysis of the 14-gene signature model in the TCGA (**A**, **B**) and ICGA (**C**, **D**) cohort. **A** and **C**. Kaplan–Meier curves for the OS of patients in the PRRIPM-high group and PRRIPM-low group in the TCGA (**A**) and ICGC (**C**) cohort. **B** and **D**. AUC of time-dependent ROC curves verified the prognostic performance of the risk score in the TCGA (**B**) and ICGC (**D**) cohort. PRRIPM, platinum resistance-related immune prognostic model

LASSO Cox regression analysis was applied to establish a platinum resistance-related immune prognostic model (PRRIPM) based on the expression profile of the above 30 genes. A 14-gene PRRIPM was constructed according to the optimal value of λ (Supplementary Fig. 1A and B). The risk score was calculated as follows: e ^(0.502 * expression level of CD40LG + 1.019 * expression level of CXCL12 + 1.197 * expression level of DUOXA1 + 1.039 * expression level of IGF2BP1 + 0.742 * expression level of IL24 + 1.018 * expression level of KMT2B + 1.098 * expression level of LIG3 + 1.117 * expression level of LIN28B + 0.881 * expression level of MAPK8 + 1.068 * expression level of RAD52 + 1.061 * expression level of STAT5A + 0.997 * expression level of TAP1 + 1.009 * expression level of TGM2 + 0.957 * expression level of TRIM27)^. The patients were stratified into a PRRIPM-high group (*n* = 113) or a PRRIPM-low group (*n* = 115) based on the median cut-off value (Supplementary Fig. 2A). Compared to the patients in the PRRIPM-low groups, the patients in the PRRIPM-high group had a significantly worse OS using the Kaplan–Meier curve (Fig. 3A , *P* < 0.0001). The predictive performance of the risk score for OS was calculated by time-dependent ROC curves, and the area under the curve (AUC) reached 0.767 at 3 years and 0.765 at 5 years (Fig. 3B). To test the robustness of the PRRIPM constructed from the TCGA cohort, the patients from the ICGC cohort were also categorized into PRRIPM-high (*n* = 41) or PRRIPM-low (*n* = 41) groups by the median value calculated with the same formula as that from the TCGA cohort (Supplementary Fig. 2B). Similar to the results obtained from the TCGA cohort, patients in the PRRIPM-high group had a reduced survival time compared with those in the PRRIPM-low group (Fig. 3C , *P* < 0.0001). Likewise, the AUC of the 14-gene PRRIPM was 0.825 at 3 years and 0.873 at 5 years (Fig. 3D).

### Immune Status Analysis in the TCGA and ICGC Cohorts

**Fig. 4:**
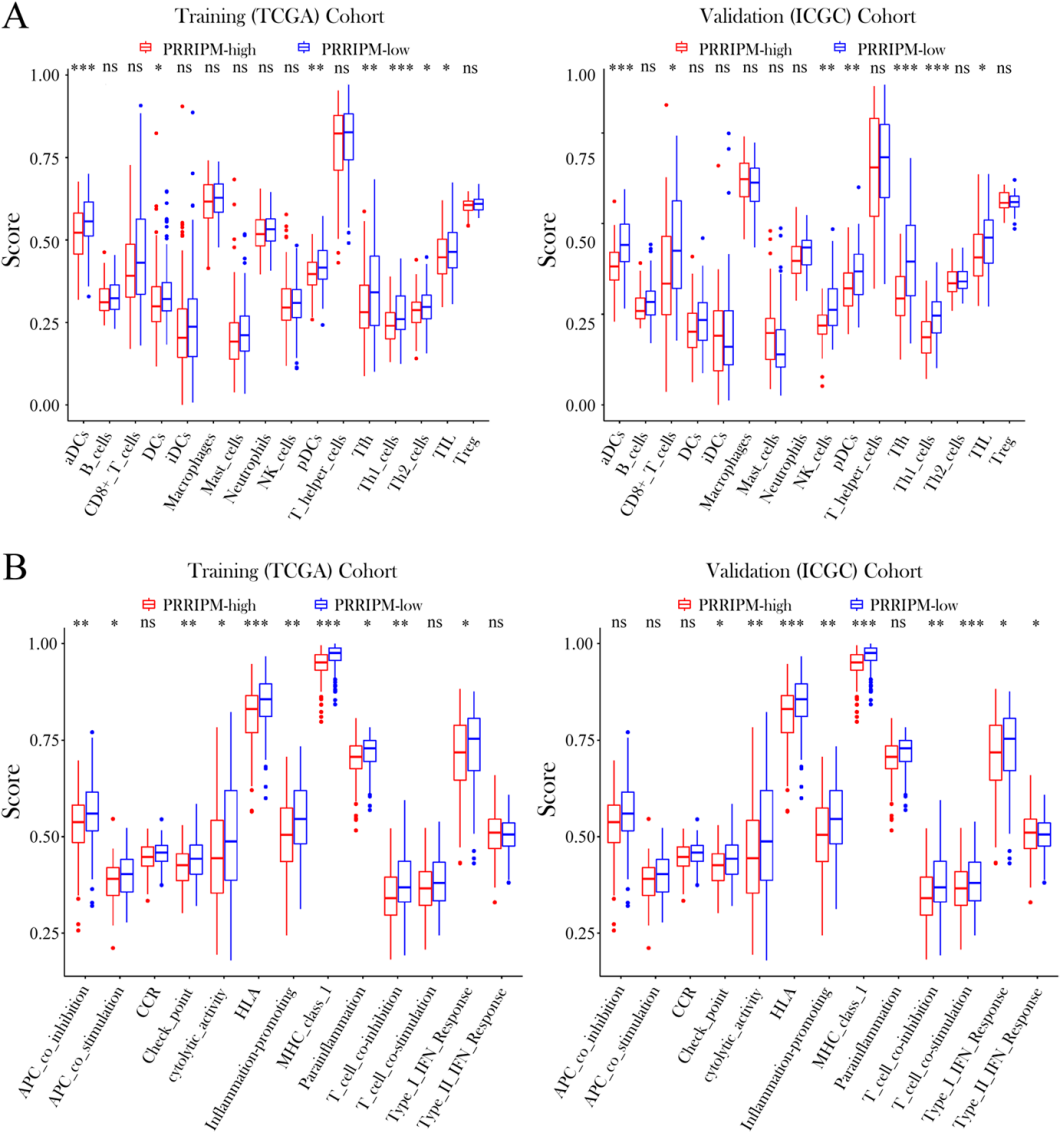
Comparison of the ssGSEA scores between different risk groups in the training cohort and validation cohort. The scores of 16 immune cells (**A**) and 13 immune-related functions (**B**) are displayed in boxplots. Tfh, T follicular helper. TIL, tumor-infiltrating lymphocyte. CCR, cytokine-cytokine receptor. *P* values were shown as: ns, not significant; *P < 0.05; **P < 0.05; ***P < 0.001

To discover the relationship between the immune status and risk score, ssGSEA was used to quantify the enrichment scores of diverse immune cell subpopulations and corresponding functions. Interestingly, the function of the antigen presentation process, including the scores of DCs, aDCs, pDCs, APC costimulation, HLA, and MHC class I, was significantly different between the PRRIPM-low and PRRIPM-high groups in the TCGA cohort (*P* < 0.05, Fig. 4A, B). Moreover, the scores of T follicular helper (Tfh) cells, Th1 cells, Th2 cells, and tumor-infiltrating lymphocyte (TIL) cells, as well as APC coinhibition, checkpoint, cytolytic activity, inflammation promotion, parainflammation, T-cell coinhibition, and type I IFN response were lower in the PRRIPM-high group (*P* < 0.05, Fig. 4A, B). Comparisons in the ICGC cohort demonstrated the differences in aDCs, CD8^+^ T cells, NK cells, pDCs, Tfh cells, Th1 cells, and TILs, as well as the checkpoint, cytolytic activity, HLA, inflammation promotion, MHC class I, T-cell coinhibition, T-cell costimulation, type I IFN response, and type II IFN response (*P* < 0.05, Fig. 4A, B). Particularly, the scores for the antigen presentation process were the most significantly different between the two risk groups in both the TCGA and ICGC cohorts.

## Discussion

In our study, the expression of 912 platinum resistance-related genes was systematically investigated in OV tumor tissues and their associations with immune score and prognosis. A novel prognostic model integrating 14 platinum resistance-related genes was first built and validated in an external cohort. Immune status analyses revealed that the antigen presentation process was involved.

Although several previous studies have revealed that a few genes might regulate platinum resistance in OV (Li et al. 2020; Wu et al. 2020; Zhang et al. 2021), their correlation with the immune score and prognosis of OV patients is poorly understood. Surprisingly, 41.1% of platinum resistance-related genes were differentially expressed between high and low immune score OV patients, and 30 of them were associated to OS in the univariate Cox regression analysis. These results confirmed the potential value of platinum resistance in the immune status of OV and the possibility of constructing a prognostic model with these platinum resistance-related immune genes.

14 platinum resistance-related immune genes (*CD40LG*, *CXCL12*, *DUOXA1*, *IGF2BP1*, *IL24*, *KMT2B*, *LIG3*, *LIN28B*, *MAPK8*, *RAD52*, *STAT5A*, *TAP1*, *TGM2*, and *TRIM27*) composed the prognostic model proposed in this study. The ligand CD40LG and its CD40 receptor are some of the most critical molecular pairs of the stimulatory immune checkpoints (Tang et al. 2021). Gallagher et al. found that the activation of the CD40LG/CD40 pathway suppressed the growth of ovarian cancer cells but promoted their apoptosis (Gallagher et al. 2002). CXCL12 is an alpha-chemokine ligand specific for the CXCR4 receptor, and the CXCL12/CXCR4 axis was found to be activated by cancer-associated fibroblasts to promote epithelial–mesenchymal transition and cisplatin resistance in ovarian cancer (Zhang et al. 2020). DUOX1, an NADPH oxidase family member, catalyzes the production of hydrogen peroxide (Sun 2020). It has been observed that the antitumor effect of DUOX1-deficient macrophages was associated with a significant increase in IFNγ production by both lymphoid and myeloid immune cells (Meziani et al. 2020). IGF2BP1 is an oncofetal RNA-binding protein that plays a major role in RNA transport, translation, and stability (Massironi et al. 2017). The protein promotes a mesenchymal tumor cell phenotype characterized by altered actin dynamics, elevated migration, invasion, proliferation, self-renewal, and anoikis resistance (Stöhr et al. 2012; Zirkel et al. 2013; Busch et al. 2016). IL24 was originally discovered to harbor a specific anticancer activity without affecting normal cells (Menezes et al. 2018), including apoptosis induction, anti-angiogenesis mechanisms, halting invasion, cancer stem cell elimination, radio-sensitization, chemo-sensitization and immune tolerance (Ma et al. 2016; Panneerselvam et al. 2013; Su et al. 2003). KMT2B, encoding a histone H3 lysine 4 (H3K4)-specific N-methyltransferase responsible for posttranslational modification of histones, plays an essential role in the regulation of dystonia (Meyer et al. 2017). Tomkinson et al. found that LIG3 could encode mitochondrial DNA ligase IIIα, which is required for mitochondrial function (Tomkinson and Sallmyr 2013). LIN28B is a highly conserved RNA-binding protein that is frequently upregulated in various cancers including ovarian cancer (Viswanathan et al. 2009), contributing to cell death resistance, tumor-associated inflammation, genome instability, acquisition of immortality, and evasion of immune destruction (Wang et al. 2015). MAPK8, also known as JNK1, positively regulates autophagy to counteract apoptosis, and its effect on autophagy is related to the development of chemotherapeutic resistance (Vasilevskaya et al. 2016) and immune evasion (Deng et al. 2011). RAD52, an important protein for homologous recombination, was found to have an important role in genomic stability maintenance and cancer suppression in mammalian cells (Nogueira et al. 2019). STAT5 provides its oncogenic activity through transcriptional alterations or protein–protein interactions, inducing anti-apoptotic signaling, aberrant DNA damage response and invasion, metastasis, and epithelial to mesenchymal transition (EMT) (Dorritie et al. 2014). TAP1, as a membrane-associated protein, plays a crucial role in transporting peptides into phagosomes and endosomes during cross-presentation in DCs (Barbet et al. 2021). TGM2 is a multifunctional transamidating acyltransferase that catalyzes calcium-dependent posttranslational modifications inducing protein cross-linking, glutamine deamination, polyamine incorporation, and guanosine triphosphate hydrolysis (Lee et al. 2021). TGM2 was found to be elevated in malignant tumors such as breast, colon, and ovarian cancers (Budillon et al. 2013). TRIM27, which belongs to the superfamily of zinc finger proteins, was previously reported as a transcriptional repressor for suppressing cell senescence, and its high expression could lead to oncogenesis and chemoresistance in multiple types of cancers including OV (Xing et al. 2020). However, whether these genes play a role in OV patient prognosis by influencing the process of platinum resistance-related immune status remains to be elucidated.

Although the mechanisms underlying tumor susceptibility to platinum-based chemotherapy have been an intense area of research in recent years, the potential modulation between tumor immunity and platinum-based chemotherapy remains elusive. In our study, we found that the contents of the antigen presentation process were significantly different between the PRRIPM-low and PRRIPM-high groups in both the TCGA and ICGC cohorts. One possible speculation is that platinum-related chemotherapy will trigger tumor starvation, destruction of tumor cells, and subsequent release of cell debris and tumor neo-antigens that are ingested by antigen-presenting cells (Macpherson et al. 2020). In addition, the PRRIPM-high groups in both the TCGA and ICGC cohorts had lower fractions of TILs and decreased type I IFN responses. Previous studies have demonstrated that decreased TILs and type I IFN responses are related to poor prognosis in OV patients due to their role in antitumor immunity (Laumont et al. 2021; Dangaj et al. 2019; Kroeger et al. 2016). Therefore, the attenuated antitumor immunity may be an explanation for poor prognosis of OV patients with high risk.

There are some limitations of this study. First, this prognostic model was both built and validated with retrospective data from public databases. More prospective real-world data are need to prove its clinical utility. Second, the intrinsic weakness of merely considering a single hallmark to construct a prognostic model was inevitable, because many prominent prognostic genes in OV might have been excluded. Additionally, it should be indicated that the association between the immune activity and risk score has not yet been experimentally verified.

Overall, this study constructed a novel prognostic model of 14 platinum resistance-related immune genes. This model correlated to OS in both the derivation and validation cohorts, providing insight into the prediction of OV clinical prognosis. The underlying mechanisms between platinum resistance-related genes and tumor immunity in OV are largely unknown and need further exploration.

## Conclusion

In conclusion, we constructed a novel platinum resistance-related immune gene signature that could predict the prognosis of OV patients. Functional analysis revealed that the attenuated antitumor immunity in patients with high risk was associated with their poor prognosis. Targeting tumor immunity may be a therapeutic alternative for OV with platinum resistance.

### Supplementary Information

Below is the link to the electronic supplementary material.

Supplementary file1 (DOCX 338 KB)

## Data Availability

The data and materials can be obtained by contacting the corresponding author.

## Abbreviations

OV: Ovarian Cancer
LASSO: Least Absolute Shrinkage and Selection Operator
OS: Overall Survival
DEGs: Differentially Expressed Genes
ROC: Receiver Operating Characteristic
TAMs: Tumor-Associated Macrophage
TIL: Tumor-Infiltrating Lymphocyte
Tfh: T follicular helper
RNA-seq: RNA Sequencing
FDR: False Discovery Rate
BH: Benjamini & Hochberg
AUC: Area Under the Curve
ssGSEA: Single-Sample Gene Set Enrichment Analysis
PRRIPM: Platinum Resistance-Related Immune Prognostic Model
EMT: Epithelial-to-Mesenchymal Transition
