# Supplementary material for: A Novel Platinum Resistance-Related Immune Gene Signature for Overall Survival Prediction in Patients with Ovarian Cancer
Source: Biochem Genet. 2023 Jun 4;62(1):112–24. doi: 10.1007/s10528-023-10379-9 (PMC10901932; doi:10.1007/s10528-023-10379-9)
Supplement: Supplementary file 1 — Supplementary file1 (DOCX 338 KB) [file 10528_2023_10379_MOESM1_ESM.docx]

**Supplementary Figure**


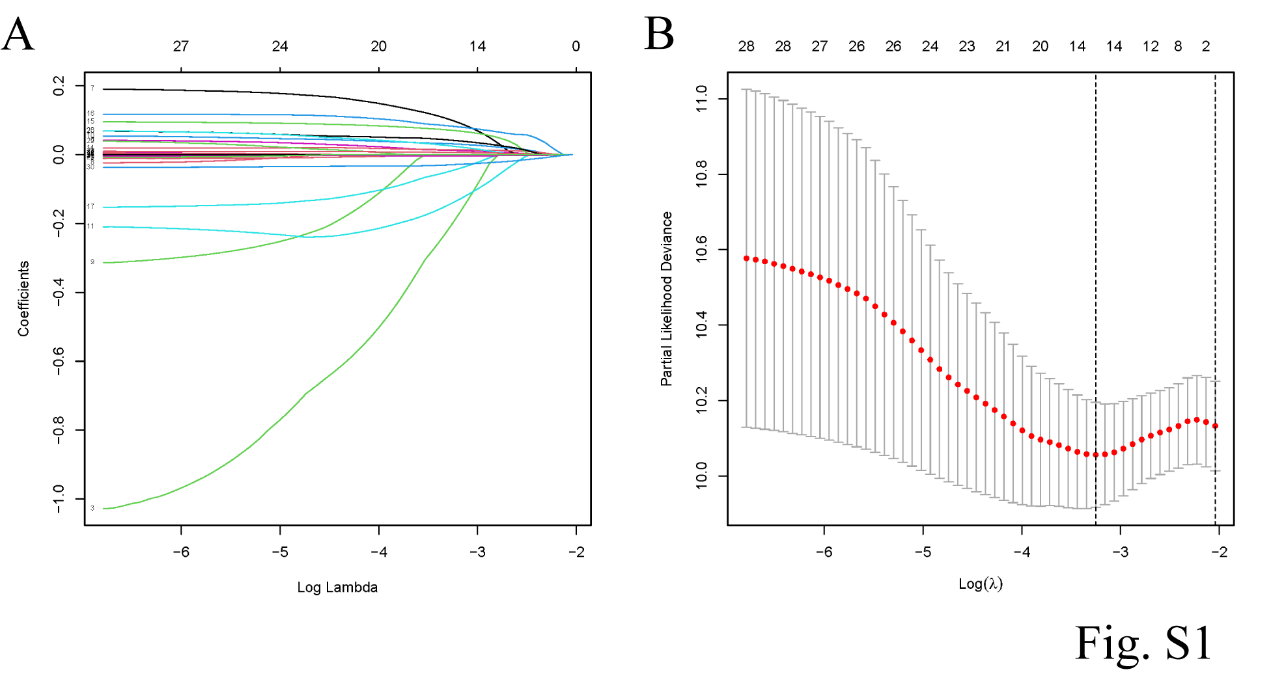


Supplementary Figure S1: Construction of a 14-gene signature model in the TCGA cohort. A. LASSO coefficient profiles of the expression 30 candidate genes. B. Selection of the penalty parameter (λ) in the LASSO model via 10-fold cross-validation. The dotted vertical lines are plotted at the optimal values following the minimum criteria (left) and “one standard error” criteria (right).


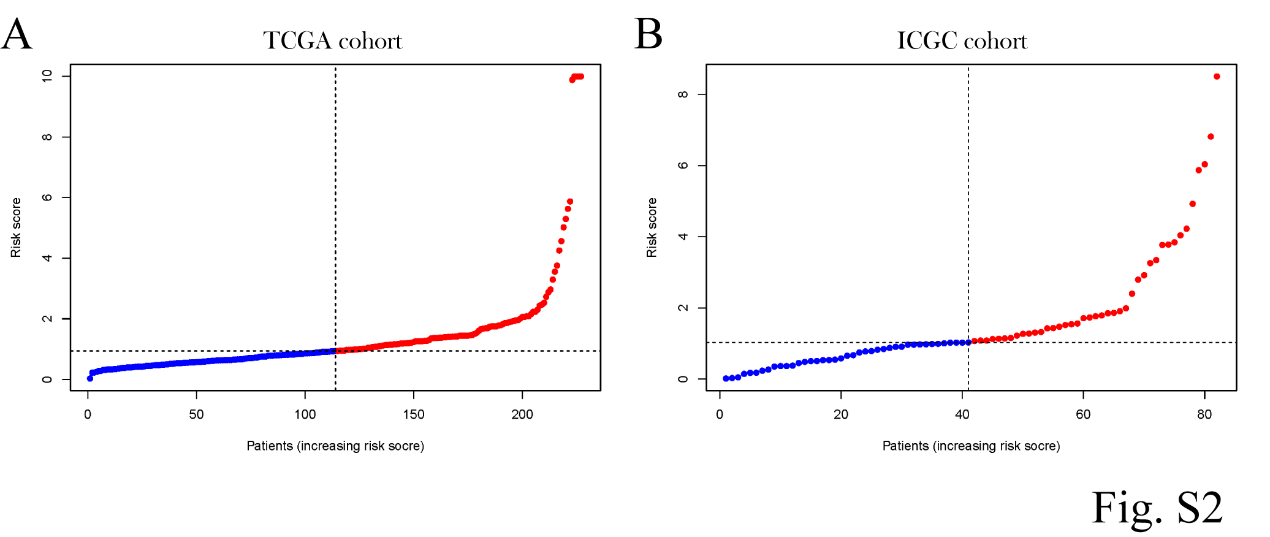


Supplementary Figure S2: The distribution and median value of the risk scores in the TCGA (a) and ICGC (b) cohort.

**Supplementary Table S1. 912 platinum resistance-related genes**

| AATF | CDCP1 | FOSB | LRRC8A | PODXL | SMO |
| --- | --- | --- | --- | --- | --- |
| ABCA8 | CDH1 | FOSL1 | LRRC8D | POLB | SMPD1 |
| ABCB1 | CDH2 | FOXC2 | LRRFIP1 | POLD1 | SMURF2 |
| ABCB5 | CDK12 | FOXM1 | LTB4R2 | POLD2 | SNAI1 |
| ABCC1 | CDK2 | FOXO1 | LUC7L3 | POLD3 | SNAI2 |
| ABCC2 | CDK4 | FOXO3 | MACC1 | POLE3 | SOD1 |
| ABCC3 | CDK5 | FOXP1 | MAD2L2 | POLH | SOD2 |
| ABCC4 | CDK7 | FSTL1 | MAFG | POLK | SOX1 |
| ABCC5 | CDKN1A | FUBP1 | MAL | POLM | SOX10 |
| ABCD2 | CDKN1B | FZD8 | MALT1 | POLN | SOX17 |
| ABCF2 | CDKN1C | G6PD | MAP1LC3A | POLQ | SOX2 |
| ABCG2 | CDKN2A | GABPA | MAP1LC3B | POSTN | SOX8 |
| ABHD2 | CDKN2C | GADD45A | MAP2K1 | POU5F1 | SOX9 |
| ABL1 | CEBPB | GADD45GIP1 | MAP2K3 | PPARGC1A | SPARC |
| ABRAXAS2 | CFLAR | GAS6 | MAP2K4 | PPARGC1B | SPINK7 |
| ACSS2 | CHD4 | GCLC | MAP3K1 | PPL | SPP1 |
| ACTA2 | CHEK1 | GCLM | MAP3K5 | PPM1D | SQSTM1 |
| ACTN4 | CHEK2 | GDF15 | MAP3K7 | PPP2CA | SRC |
| ACVR1C | CHMP2B | GFRA1 | MAPK1 | PPP2R2A | SREBF2 |
| ADAM10 | CHUK | GGNBP2 | MAPK11 | PRDX3 | SRPK1 |
| ADAM17 | CIP2A | GGT1 | MAPK12 | PRKAA1 | SRPK2 |
| ADAM9 | CLDN1 | GJA1 | MAPK13 | PRKAA2 | SRSF2 |
| AGR3 | CLDN3 | GLI1 | MAPK14 | PRKAB1 | SRSF4 |
| AIFM1 | CLDN4 | GLI2 | MAPK3 | PRKAB2 | ST6GAL1 |
| AKR1B10 | CLDN7 | GLS | MAPK8 | PRKAR1A | STAT1 |
| AKR1C1 | CLIC1 | GLS2 | MAPK9 | PRKCA | STAT3 |
| AKR1C2 | CLN3 | GNPTG | MAST1 | PRKCB | STAT5A |
| AKR1C3 | CLOCK | GOLPH3 | MBNL1 | PRKCD | STAT5B |
| AKT1 | CLPTM1L | GOLPH3L | MCL1 | PRKDC | STAT6 |
| AKT1S1 | CLU | GPBP1 | MCM8 | PRKG1 | STIM1 |
| AKT2 | COL11A1 | GPRC5A | MCM9 | PRKRA | STING1 |
| ALDH1A1 | COL1A1 | GPX3 | MDM2 | PROM1 | STK11 |
| ALDH3A1 | COL3A1 | GPX4 | MDM4 | PRR13 | STK17A |
| ALDOA | COL6A3 | GREB1 | MELK | PRSS1 | STK4 |
| ALKBH3 | COX17 | GREM1 | MEN1 | PSMB10 | SUSD2 |
| AMBRA1 | COX7A2 | GSK3B | MET | PSMB9 | TAB1 |
| AMFR | CPT2 | GSN | METTL6 | PTAFR | TAB3 |
| ANKRD1 | CREB1 | GSR | MGAT1 | PTCH1 | TAP1 |
| ANXA2 | CRYAB | GSS | MGAT5 | PTEN | TBCE |
| ANXA3 | CSF1 | GSTA1 | MGMT | PTGER3 | TBX2 |
| ANXA4 | CSF1R | GSTK1 | MICU1 | PTGS2 | TEAD1 |
| APAF1 | CTNNA1 | GSTM1 | MIEN1 | PTK2 | TEAD3 |
| APC | CTNNB1 | GSTM4 | MITF | PTPN3 | TEAD4 |
| APEX1 | CUEDC2 | GSTP1 | MLH1 | PXN | TERT |
| AQP2 | CUL3 | GSTT1 | MME | RAB18 | TET1 |
| AREG | CUL4A | H2AX | MMP10 | RAB25 | TET2 |
| ARHGDIB | CUZD1 | H6PD | MMP19 | RAB5C | TFAM |
| ARID1A | CXCL10 | HBEGF | MMP2 | RAB7A | TFRC |
| ARID3B | CXCL12 | HCFC1R1 | MMP20 | RAB8A | TGFB1 |
| ARIH1 | CXCL8 | HDAC1 | MMP7 | RAC1 | TGFB2 |
| ARL6IP5 | CXCR4 | HDAC3 | MMP9 | RAD23B | TGFB3 |
| ARNT | CYP1B1 | HDAC4 | MNAT1 | RAD50 | TGFBI |
| ASS1 | CYP2E1 | HECTD3 | MOAP1 | RAD51 | TGFBR1 |
| ATF2 | DACT1 | HELQ | MRE11 | RAD51B | TGFBR2 |
| ATF3 | DAPK1 | HEY1 | MSH2 | RAD51C | TGM2 |
| ATF4 | DAPK3 | HGF | MSH3 | RAD51D | TGM3 |
| ATF6 | DAXX | HIC1 | MSH5 | RAD52 | THBS1 |
| ATG12 | DCLRE1A | HIF1A | MSH6 | RAD54L | TIE1 |
| ATG14 | DCLRE1B | HIPK2 | MSLN | RADX | TIGAR |
| ATG5 | DCLRE1C | HK2 | MSX1 | RASSF1 | TIMELESS |
| ATG7 | DDB1 | HMGB1 | MT1A | RB1 | TIMP2 |
| ATM | DDB2 | HMGB2 | MT2A | RBBP8 | TIMP3 |
| ATMIN | DDIAS | HMGCR | MT3 | RBM17 | TLK1 |
| ATP11B | DDIT3 | HMOX1 | MT-CO3 | RBM3 | TLR4 |
| ATP1A1 | DDR1 | HNF1B | MTDH | RBMS3 | TLX3 |
| ATP1B1 | DERL1 | HOXA13 | MTOR | RECQL | TMEM205 |
| ATP6V0B | DHFR | HOXA4 | MTRR | RECQL4 | TMEM88 |
| ATP6V0D1 | DIAPH3 | HOXB13 | MUC1 | RELA | TMEM97 |
| ATP6V1A | DICER1 | HOXB3 | MUC16 | RELB | TMEM98 |
| ATP6V1B2 | DIRAS3 | HOXB4 | MUC5B | RET | TNF |
| ATP6V1C1 | DKK1 | HOXB7 | MUS81 | REV1 | TNFAIP8 |
| ATP6V1E1 | DKK3 | HOXD8 | MUTYH | REV3L | TNFAIP8L2 |
| ATP7A | DNMT1 | HROB | MVP | RFC1 | TNFRSF10A |
| ATP7B | DOK2 | HSF1 | MYC | RFC2 | TNFRSF10B |
| ATR | DRAM1 | HSP90AA1 | MYD88 | RGS10 | TNFRSF12A |
| ATRIP | DSG1 | HSP90AB1 | MZF1 | RGS17 | TNFSF10 |
| AURKA | DUOXA1 | HSPA1A | NACC1 | RHOA | TOP1 |
| AXL | DUSP1 | HSPA1B | NANOG | RHOJ | TOP2A |
| BAD | DUSP6 | HSPA5 | NAP1L3 | RIF1 | TOP3A |
| BAG3 | DVL2 | HSPB1 | NAPSA | RIPK1 | TOP3B |
| BAK1 | DVL3 | HSPD1 | NAV3 | RIPK3 | TOPBP1 |
| BAP1 | DYNLL1 | HTRA2 | NBN | RNASET2 | TP53 |
| BARD1 | DYRK2 | ICAM1 | NCOA3 | RNF2 | TP53BP1 |
| BAX | E2F1 | ID1 | NDUFA13 | ROR1 | TP53INP1 |
| BBC3 | E2F2 | IFNG | NEDD4 | ROR2 | TP63 |
| BCAT1 | E2F4 | IGF1 | NEDD4L | RORC | TP73 |
| BCL2 | E2F7 | IGF1R | NEDD8 | RPA1 | TPT1 |
| BCL2A1 | EDNRA | IGF2 | NEIL2 | RPA2 | TRAF2 |
| BCL2L1 | EGF | IGF2BP1 | NEK11 | RPL36 | TRAP1 |
| BCL2L11 | EGFR | IGF2BP3 | NES | RPL37 | TRIB2 |
| BCL2L2 | EGLN1 | IGF2R | NF1 | RPS20 | TRIM27 |
| BECN1 | EGLN3 | IKBKB | NF2 | RPS4X | TRIM65 |
| BEX3 | EGR1 | IKBKE | NFE2L2 | RPS6KA1 | TRIP13 |
| BHLHE40 | EHD1 | IL11 | NFKB1 | RPS6KA3 | TRO |
| BID | EIF2AK3 | IL17A | NFKB2 | RPS6KB1 | TUBB3 |
| BIK | EIF2S1 | IL1A | NID1 | RPS6KB2 | TWIST1 |
| BIN1 | EIF3A | IL1B | NKX2-8 | RPS7 | TXN |
| BIRC2 | EIF3G | IL22 | NOS1 | RPSA | TXNDC17 |
| BIRC3 | EIF4E | IL24 | NOS2 | RRM2 | TXNL1 |
| BIRC5 | EIF4EBP1 | IL25 | NOS3 | RUNX3 | TXNRD1 |
| BIRC7 | EIF4G2 | IL6 | NOTCH1 | S100A4 | TXNRD2 |
| BLM | EME1 | IL6R | NOTCH3 | S100A7 | TXNRD3 |
| BMI1 | EMSY | IL7 | NOX5 | S100A9 | UBE2S |
| BMP4 | EMX2 | ILK | NPM1 | S100B | UBE2T |
| BMPR1A | ENG | IRF1 | NQO1 | SAT1 | UBR5 |
| BNIP3 | ENO1 | ITGA5 | NR1I2 | SAV1 | UCHL1 |
| BOK | ENPP2 | ITGA6 | NR4A1 | SCAI | UCP2 |
| BRCA1 | EP300 | ITGB1 | NRAS | SCRIB | UGCG |
| BRCA2 | EPCAM | ITGB5 | NRP2 | SDHB | UIMC1 |
| BRD9 | EPHA2 | ITGB6 | NT5E | SDHC | UNG |
| BRIP1 | ERBB2 | ITGB8 | NTHL1 | SDHD | URI1 |
| BSG | ERBB3 | ITM2A | NTRK3 | SENP1 | USP1 |
| C1QBP | ERBB4 | ITPR1 | NTS | SERPINB2 | USP13 |
| CAD | ERCC1 | JAG1 | NTSR1 | SERPINB3 | USP22 |
| CAMK2D | ERCC2 | JAK2 | NUP62 | SERPINE1 | USP35 |
| CAPN1 | ERCC3 | JUN | ORAI1 | SESN1 | USP37 |
| CAPNS1 | ERCC4 | JUNB | P4HB | SETD2 | UVRAG |
| CARD10 | ERCC5 | JUND | PAK1 | SFN | VCAM1 |
| CASP12 | ERCC6 | KAT2B | PAK5 | SFRP5 | VDAC1 |
| CASP14 | ERCC8 | KAT5 | PDIA3 | SGK1 | VEGFA |
| CASP2 | ERN1 | KAT8 | PDK1 | SHC1 | VHL |
| CASP3 | ETS1 | KCNMA1 | PDK4 | SHCBP1 | VIM |
| CASP4 | EXO1 | KDM3A | PDPK1 | SIK2 | WDR48 |
| CASP6 | EZH2 | KDM5A | PDXK | SIRT1 | WFDC2 |
| CASP7 | EZR | KDR | PDXP | SIRT3 | WWOX |
| CASP8 | FANCB | KEAP1 | PDZK1 | SIX1 | WWP2 |
| CASP9 | FANCC | KIF20A | PEA15 | SLC19A1 | WWTR1 |
| CAV1 | FANCD2 | KISS1 | PER2 | SLC1A5 | XAF1 |
| CAVIN3 | FANCE | KIT | PFKFB3 | SLC22A1 | XBP1 |
| CBS | FANCF | KLF4 | PGD | SLC22A2 | XIAP |
| CCDC69 | FANCG | KLF5 | PGK1 | SLC22A3 | XPA |
| CCL2 | FANCI | KMT2B | PGRMC1 | SLC25A1 | XPC |
| CCL25 | FANCL | KMT2C | PHB | SLC27A2 | XPO1 |
| CCL5 | FANCM | KRAS | PHGDH | SLC2A1 | XRCC1 |
| CCN1 | FAP | KRT1 | PIK3CA | SLC2A14 | XRCC2 |
| CCN2 | FAS | KRT10 | PIK3CB | SLC31A1 | XRCC3 |
| CCNA2 | FASLG | KRT18 | PIK3CG | SLC31A2 | XRCC4 |
| CCND1 | FASN | KRT5 | PIK3R1 | SLC39A4 | XRCC5 |
| CCNE1 | FAT1 | KRT8 | PIK3R2 | SLC3A2 | XRCC6 |
| CCNI | FAU | L1CAM | PIM1 | SLC40A1 | YAP1 |
| CCR9 | FBXO32 | LAMTOR5 | PIM2 | SLC46A1 | YBX1 |
| CD24 | FEN1 | LATS1 | PINK1 | SLC7A11 | YTHDF1 |
| CD274 | FGF1 | LCK | PIWIL2 | SLC7A5 | YWHAG |
| CD40 | FGF2 | LDLR | PKM | SLC9A3R1 | YWHAH |
| CD40LG | FGFR2 | LGALS3 | PLAA | SLCO1B3 | YWHAQ |
| CD44 | FH | LIG3 | PLK1 | SLFN11 | YWHAZ |
| CD55 | FLNA | LIG4 | PLK2 | SLX1A | YY1 |
| CD8A | FLT4 | LIN28A | PMAIP1 | SLX4 | ZBTB7A |
| CD8B | FN1 | LIN28B | PMEL | SMAD4 | ZEB1 |
| CDC37 | FOLR1 | LMO4 | PML | SMARCA2 | ZEB2 |
| CDC42 | FOLR2 | LNPEP | PMS2 | SMARCA4 | ZNF143 |
| CDC7 | FOS | LRP1 | PNKP | SMARCE1 | ZNF93 |

**Supplementary Table S2**. The annotated gene set file used in ssGSEA

| 1. aDCs | | | | | |
| --- | --- | --- | --- | --- | --- |
| C10orf54 | CD274 | LGALS9 | PDCD1LG2 | PVRL3 |  |
| 1. B_cells | | | | | |
| BACH2 | BANK1 | BLK | BTLA | CD79A | CD79B |
| FCRL1 | FCRL3 | HVCN1 | RALGPS2 |  |  |
| 1. CD8+_T_cells | | | | | |
| CD8A |  |  |  |  |  |
| 1. DCs | | | | | |
| CCL17 | CCL22 | CD209 | CCL13 |  |  |
| 1. iDCs | | | | | |
| CD1A | CD1E |  |  |  |  |
| 1. Macrophages | | | | | |
| C11orf45 | CD68 | CLEC5A | CYBB | FUCA1 | GPNMB |
| HS3ST2 | LGMN | MMP9 | TM4SF19 |  |  |
| 1. Mast_cells | | | | | |
| CMA1 | MS4A2 | TPSAB1 |  |  |  |
| 1. Neutrophils | | | | | |
| EVI2B | HSD17B11 | KDM6B | MEGF9 | MNDA | NLRP12 |
| PADI4 | SELL | TRANK1 | VNN3 |  |  |
| 1. NK_cells | | | | | |
| KLRC1 | KLRF1 |  |  |  |  |
| 1. pDCs | | | | | |
| CLEC4C | CXCR3 | GZMB | IL3RA | IRF7 | IRF8 |
| LILRA4 | PHEX | PLD4 | PTCRA |  |  |
| 1. T_helper_cells | | | | | |
| CD4 |  |  |  |  |  |
| 1. Tfh | | | | | |
| PDCD1 | CXCL13 | CXCR5 |  |  |  |
| 1. Th1_cells | | | | | |
| IFNG | TBX21 | CTLA4 | STAT4 | CD38 | IL12RB2 |
| LTA | CSF2 |  |  |  |  |
| 1. Th2_cells | | | | | |
| PMCH | LAIR2 | SMAD2 | CXCR6 | GATA3 | IL26 |
| 1. TIL | | | | | |
| ITM2C | CD38 | THEMIS2 | GLYR1 | ICOS | F5 |
| TIGIT | KLRD1 | IRF4 | PRKCQ | FCRL5 | SIRPG |
| LPXN | IL2RG | CCL5 | LCK | TRAF3IP3 | CD86 |
| MAL | LILRB1 | DOK2 | CD6 | PAG1 | LAX1 |
| PLEK | PIK3CD | SLAMF1 | XCL1 | GPR171 | XCL2 |
| TBX21 | CD2 | CD53 | KLHL6 | SLAMF6 | CD40 |
| SIT1 | TNFRSF4 | CD79A | CD247 | LCP2 | CD3D |
| CD27 | SH2D1A | FYB | ARHGAP30 | ACAP1 | CST7 |
| CD3G | IL2RB | CD3E | FCRL3 | CORO1A | ITK |
| TCL1A | CYBB | CSF2RB | IKZF1 | NCF4 | DOCK2 |
| CCR2 | PTPRC | PLAC8 | NCKAP1L | IL7R | CD28 |
| STAT4 | CD8A | LY9 | CD48 | HCST | PTPRCAP |
| SASH3 | ARHGAP25 | LAT | TRAT1 | IL10RA | PAX5 |
| CCR7 | DOCK11 | PARVG | SPNS1 | CD52 | HCLS1 |
| ARHGAP9 | GIMAP6 | PRKCB | MS4A1 | GPR18 | TBC1D10C |
| GVINP1 | P2RY8 | EVI2B | VAMP5 | KLRK1 | SELL |
| MPEG1 | MS4A6A | ARHGAP15 | MFNG | GZMK | SELPLG |
| TARP | GIMAP7 | FAM65B | INPP5D | ITGA4 | MZB1 |
| GPSM3 | STK10 | CLEC2D | IL16 | NLRC3 | GIMAP5 |
| GIMAP4 | IFFO1 | CFH | PVRIG | CFHR1 |  |
| 1. Treg | | | | | |
| IL12RB2 | TMPRSS6 | CTSC | LAPTM4B | TFRC | RNF145 |
| NETO2 | ADAT2 | CHST2 | CTLA4 | NFE2L3 | LIMA1 |
| IL1R2 | ICOS | HSDL2 | HTATIP2 | FKBP1A | TIGIT |
| CCR8 | LTA | SLC35F2 | IL21R | AHCYL1 | SOCS2 |
| ETV7 | BCL2L1 | RRAGB | ACSL4 | CHRNA6 | BATF |
| LAX1 | ADPRH | TNFRSF4 | ANKRD10 | CD274 | CASP1 |
| LY75 | NPTN | SSTR3 | GRSF1 | CSF2RB | TMEM184C |
| NDFIP2 | ZBTB38 | ERI1 | TRAF3 | NAB1 | HS3ST3B1 |
| LAYN | JAK1 | VDR | LEPROT | GCNT1 | PTPRJ |
| IKZF2 | CSF1 | ENTPD1 | TNFRSF18 | METTL7A | KSR1 |
| SSH1 | CADM1 | IL1R1 | ACP5 | CHST7 | THADA |
| CD177 | NFAT5 | ZNF282 | MAGEH1 |  |  |
| 1. APC_co_inhibition | | | | | |
| C10orf54 | CD274 | LGALS9 | PDCD1LG2 | PVRL3 |  |
| 1. APC_co_stimulation | | | | | |
| CD40 | CD58 | CD70 | ICOSLG | SLAMF1 | TNFSF14 |
| TNFSF15 | TNFSF18 | TNFSF4 | TNFSF8 | TNFSF9 |  |
| 1. CCR | | | | | |
| CCL16 | IL1R1 | IL16 | IL12RB2 | CCRL2 | CCL14 |
| TPO | CXCR4 | IL1RL1 | CCL1 | IFNA10 | IFNA21 |
| TGFBR2 | CXCR2P1 | ILK | IL17RA | TNFRSF17 | TNFSF18 |
| CXCL2 | TGFB1I1 | CCL25 | CCR1 | IFNA13 | CCL8 |
| CCL14 | IFNGR1 | ILDR2 | IL1RN | IL20 | IL17RB |
| TGFBR3 | IL9R | CXCR1 | TNFRSF11B | IL18BP | TNFRSF25 |
| IL11RA | IL1RAPL1 | IL36RN | TNFRSF14 | CCL3L1 | IL22 |
| CCL11 | IL11 | IL34 | IL13 | TNFSF12 | IL10RB |
| IL4I1 | CSF1 | TGFB1 | IL2RB | IL5 | IFNAR2 |
| IL33 | IL20RA | IFNG | BMP8B | IL23R | CCL18 |
| CXCL12 | IL25 | IL19 | CCL2 | IL26 | IFNA16 |
| CXCL10 | TNFRSF4 | ILKAP | IL24 | TNF | CSF2RB |
| BMPER | IL18 | BMP2K | IL18RAP | TGFA | IL36A |
| BMP8A | ILF3 | CCR10 | TGFBI | CSF2 | TNFAIP3 |
| CXCL11 | CCL20 | ILDR1 | TNFSF10 | IL1F10 | IL13RA2 |
| IL21R | TNFRSF12A | EPO | TNFRSF11A | CXCL17 | IL13RA1 |
| IL17B | IL6ST | CCR7 | CXCL5 | TNFSF13 | CCR9 |
| TNFRSF9 | CXCL13 | IL17C | IL5RA | IFNA4 | TNFRSF10A |
| ILF2 | IL12B | IL23A | TNFSF9 | IL37 | IFNA7 |
| CX3CR1 | TNFRSF8 | CCR5 | IL1RL2 | IL12A | IFNW1 |
| CCR8 | IL6R | IL7 | TNFRSF13C | IL7R | XCL2 |
| TNFSF12 | BMPR2 | EPOR | IL36G | IFNA1 | TNFSF14 |
| CSF3 | IFNE | CCL13 | IL15RA | IL1A | CCR2 |
| TNFSF4 | IL1RAPL2 | IL2RG | TNFRSF21 | IL4 | BMP15 |
| BMP3 | IL3RA | IL31RA | CXCL8 | IL2 | BMP10 |
| CX3CL1 | BMP4 | TNFAIP6 | IL22RA2 | CCL22 | CCL15 |
| BMP5 | CCL24 | IFNL2 | TNFAIP8L2 | CSF3R | TGFBR1 |
| CXCR2 | TNFSF13B | BMP1 | IL18R1 | IL10 | IFNA5 |
| TNFRSF10D | CCR4 | IL12RB1 | IFNLR1 | IFNK | BMP7 |
| BMP2 | IL2RA | TNFAIP8 | CXCR6 | TGFB2 | IFNA14 |
| CXCL14 | IL32 | IL4R | CCL3L3 | IL1R2 | IL20RB |
| CCL28 | TNFRSF10C | TNFRSF6B | TNFRSF1A | IL1B | IL10RA |
| CXCL3 | IL22RA1 | TNFAIP8L1 | IL17RE | IL17F | IFNA17 |
| BMP6 | BMPR1A | TNFRSF10B | IFNGR2 | IL27RA | CCR6 |
| CCL21 | CXCR5 | IFNL3 | IL17RC | IL15 | TGFB3 |
| CXCL9 | CXCR3 | CCL5 | TNFAIP8L3 | TNFSF8 | CCL15 |
| CCL23 | IFNA8 | CXCL6 | ILVBL | IL36B | CCL4 |
| IL6 | IL17REL | CXCL1 | TGFBRAP1 | XCL1 | CCL27 |
| TNFRSF18 | IFNB1 | CCR3 | CCL4L1 | CXCL16 | TNFRSF13B |
| IL17RD | IFNAR1 | TNFSF11 | CSF2RA | TNFRSF19 | TNFSF13 |
| IL17D | TNFRSF1B | CSF1R | CCRN4L | IL3 | CCL14 |
| IL27 | CCL17 | IL21 | CCL26 | CCL3 |  |
| CCL7 | IFNL1 | IL1RAP | TNFAIP1 | IFNA2 |  |
| 1. Check-point | | | | | |
| IDO1 | CD70 | TNFSF15 | CD244 | CD48 | TNFRSF18 |
| LAG3 | TNFSF9 | TNFRSF14 | CD274 | TNFRSF25 | BTNL2 |
| CTLA4 | ICOSLG | IDO2 | HAVCR2 | CD40LG | C10orf54 |
| TNFRSF9 | KIR3DL1 | CD276 | CD27 | ADORA2A | CD200R1 |
| ICOS | CD86 | CD40 | BTLA | VTCN1 | TNFSF4 |
| CD80 | PDCD1 | TNFRSF4 | LGALS9 | CD160 | CD200 |
| PDCD1LG2 | LAIR1 | TNFSF14 | TMIGD2 | CD44 | NRP1 |
| TIGIT | TNFRSF8 | HHLA2 | CD28 | TNFSF18 |  |
| 1. Cytolytic_activity | | | | | |
| PRF1 | GZMA |  |  |  |  |
| 1. HLA | | | | | |
| HLA-E | HLA-DPB2 | HLA-C | HLA-J | HLA-DQB1 | HLA-DQB2 |
| HLA-DQA2 | HLA-DQA1 | HLA-A | HLA-DMA | HLA-DOB | HLA-DRB1 |
| HLA-H | HLA-B | HLA-DRB5 | HLA-DOA | HLA-DPB1 | HLA-DRA |
| HLA-DRB6 | HLA-L | HLA-F | HLA-G | HLA-DMB | HLA-DPA1 |
| 1. Inflammation-promoting | | | | | |
| CCL5 | CD19 | CD8B | CXCL10 | CXCL13 | CXCL9 |
| GNLY | GZMB | IFNG | IL12A | IL12B | IRF1 |
| PRF1 | STAT1 | TBX21 |  |  |  |
| 1. MHC_class_I | | | | | |
| B2M | HLA-A | TAP1 |  |  |  |
| 1. Parainflammation | | | | | |
| CXCL10 | ICAM1 | MX1 | SCARB1 | OAS2 | NOX1 |
| PLAT | MX2 | HMOX1 | CD14 | REL | PLA2G2A |
| CCND1 | CXCL9 | CD276 | BLNK | OAS3 | IFIT1 |
| LGMN | ANXA1 | TIRAP | IFIT3 | CD44 | IFITM3 |
| PLAUR | TLR2 | IL33 | RETNLB | PPARG | IL1RN |
| AIM2 | PLA2G2D | PTGES | IFIT2 | BST2 |  |
| MMP7 | ITGA2 | TNFRSF12A | ISG15 | OAS1 |  |
| 1. T_cell_co-inhibition | | | | | |
| BTLA | C10orf54 | CD160 | CD244 | CD274 | CTLA4 |
| HAVCR2 | LAG3 | LAIR1 | TIGIT |  |  |
| 1. T_cell_co-stimulation | | | | | |
| CD2 | CD226 | CD27 | CD28 | CD40LG | ICOS |
| SLAMF1 | TNFRSF18 | TNFRSF25 | TNFRSF4 | TNFRSF8 | TNFRSF9 |
| TNFSF14 |  |  |  |  |  |
| 1. Type_I_IFN_Reponse | | | | | |
| DDX4 | IFIT1 | IFIT2 | IFIT3 | IRF7 | ISG20 |
| MX1 | MX2 | RSAD2 | TNFSF10 |  |  |
| 1. Type_II_IFN_Reponse | | | | | |
| GPR146 | SELP | AHR |  |  |  |
